# Supplementary material for: Dietary Interventions Ameliorate Infectious Colitis by Restoring the Microbiome and Promoting Stem Cell Proliferation in Mice
Source: Int J Mol Sci. 2021 Dec 29;23(1):339. doi: 10.3390/ijms23010339 (PMC8745185; doi:10.3390/ijms23010339)

Supplementary Figure 2. Changes in the relative abundance of top 6 phyla. The relative abundance at the phylum level in fecal samples of Control (N), CR and CR+Tributyrin samples.

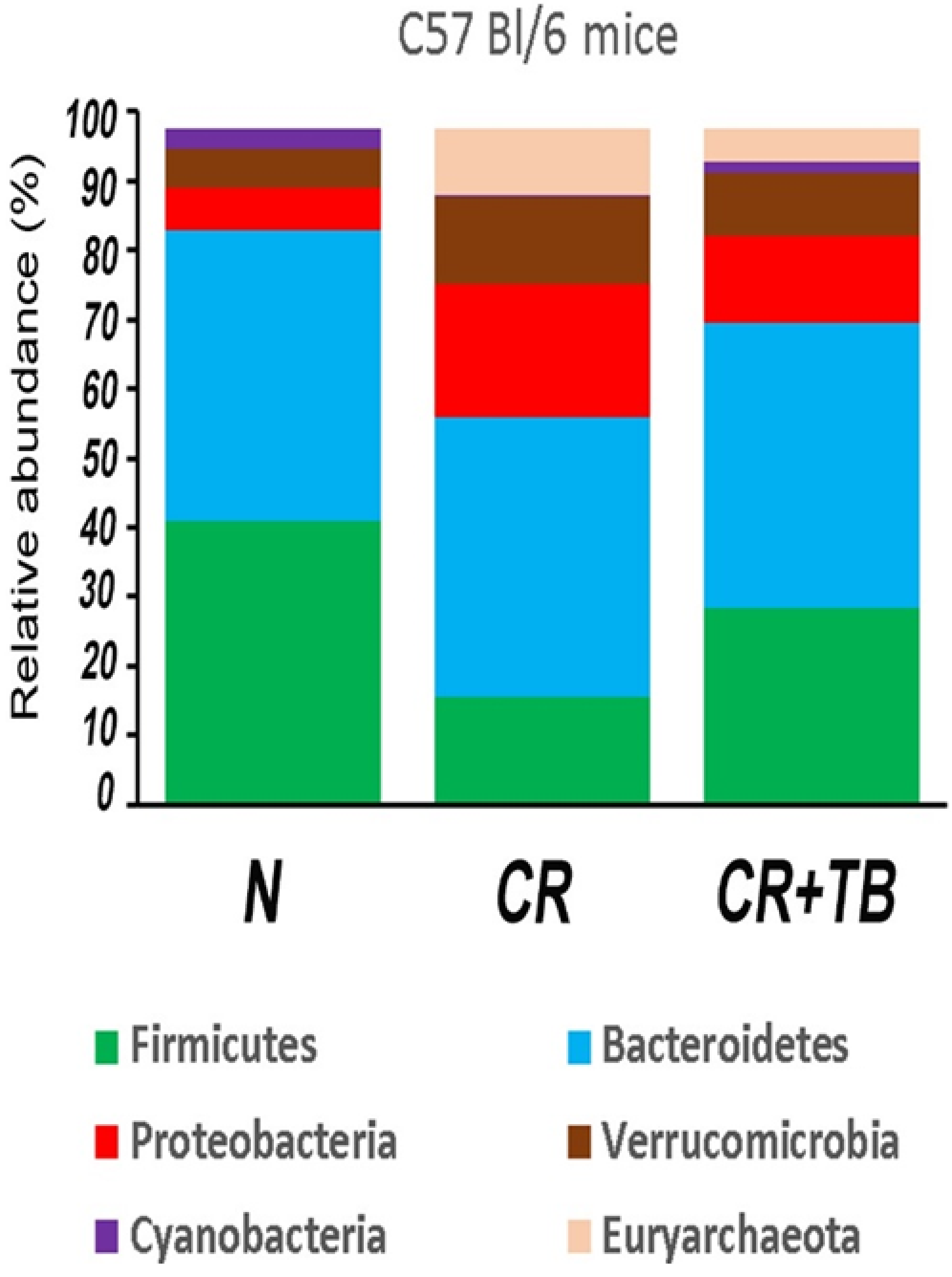

Supplement: Supplementary file 1 [file ijms-23-00339-s001.zip › Supplementary Fig 2.pdf]
