# Supplementary material for: Dietary Interventions Ameliorate Infectious Colitis by Restoring the Microbiome and Promoting Stem Cell Proliferation in Mice
Source: Int J Mol Sci. 2021 Dec 29;23(1):339. doi: 10.3390/ijms23010339 (PMC8745185; doi:10.3390/ijms23010339)
Supplement: Supplementary file 1 [file ijms-23-00339-s001.zip › Supplementary Fig 3.pdf]

Scheme 3. RNA-seq. A–C. Volcano plots showing the false discovery rate (FDR) and fold enrichment of genes differentially expressed in the control vs CR-infected groups, CR+Butyrate vs CR-infected groups and CR+B vs control groups. **D–F.** Heat maps showing a matrix layout of some selected genes and their corresponding fold change in each treatment groups.

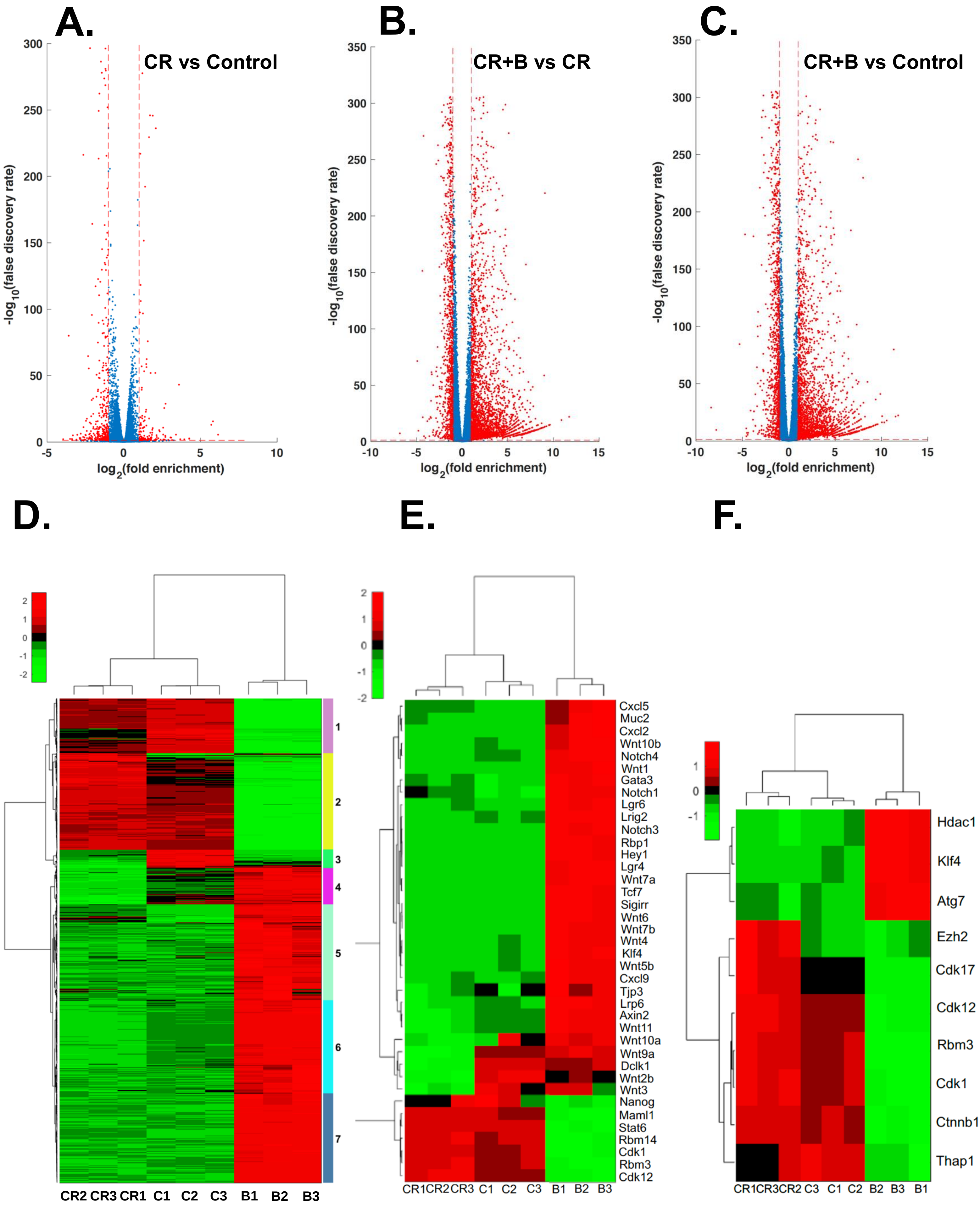
A.
CR vs Control
-log10(false discovery rate)
log2(fold enrichment)
B.
CR+B vs CR
C.
CR+B vs Control
D.
CR2 CR3 CR1 C1 C2 C3 B1 B2 B3
E.
Cxcl5
Muc2
Cxcl2
Wnt10b
Notch4
Wnt1
Gata3
Notch1
Lgr6
Lrig2
Notch3
Rbp1
Hey1
Lgr4
Wnt7a
Tcf7
Sigirr
Wnt6
Wnt7b
Wnt4
Klf4
Wnt5b
Cxcl9
Tjp3
Lrp6
Axin2
Wnt11
Wnt10a
Wnt9a
Dclk1
Wnt2b
Wnt3
Nanog
Maml1
Stat6
Rbm14
Cdk1
Rbm3
Cdk12
CR1 CR2 CR3 C1 C2 C3 B1 B2 B3
F.
Hdac1
Klf4
Atg7
Ezh2
Cdk17
Cdk12
Rbm3
Cdk1
Ctnnb1
Thap1
CR1 CR3 CR2 C3 C1 C2 B2 B3 B1
