# Supplementary material for: Dietary Interventions Ameliorate Infectious Colitis by Restoring the Microbiome and Promoting Stem Cell Proliferation in Mice
Source: Int J Mol Sci. 2021 Dec 29;23(1):339. doi: 10.3390/ijms23010339 (PMC8745185; doi:10.3390/ijms23010339)

Supplementary Figure 4. Representative bar graphs showing average number of *LacZ+* (**A,B**) or Ki-67+ cells (**C**) per crypt. Statistical analysis was performed using Two-tailed Student T-test; *$p \leq 0.05$; **$p < 0.001$.

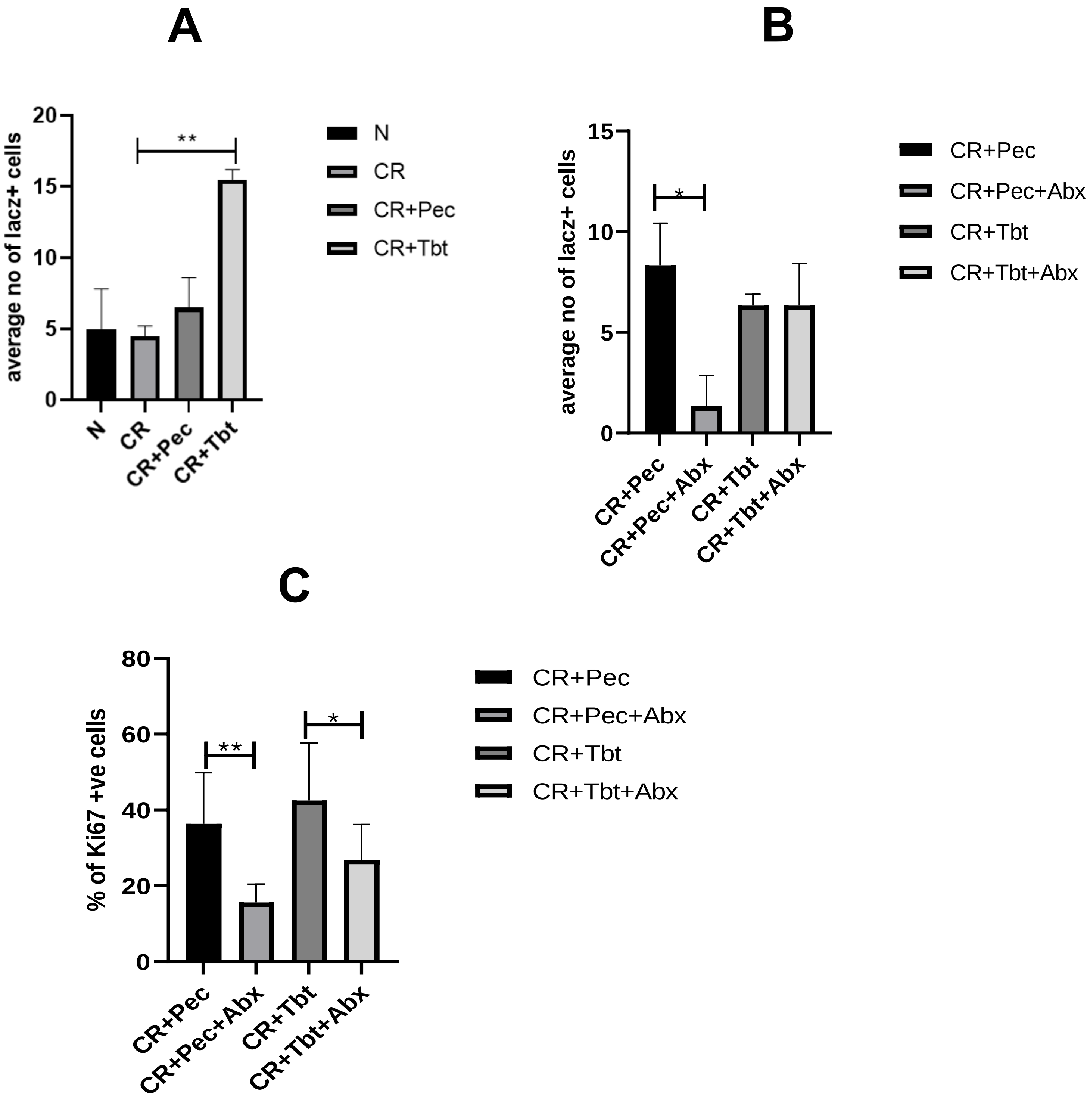

Supplement: Supplementary file 1 [file ijms-23-00339-s001.zip › Supplementary Fig 4.pdf]
