# Supplementary material for: Dietary Interventions Ameliorate Infectious Colitis by Restoring the Microbiome and Promoting Stem Cell Proliferation in Mice
Source: Int J Mol Sci. 2021 Dec 29;23(1):339. doi: 10.3390/ijms23010339 (PMC8745185; doi:10.3390/ijms23010339)
Supplement: Supplementary file 1 [file ijms-23-00339-s001.zip › Supplementary Fig 5.pdf]

Scheme 5. Chromatin immunoprecipitation-sequencing (ChIP-seq). The distribution of differential binding events of H3K27Ac, H3K4Me3 and H3K9Ac marker compared across N, CR and CR-B groups, respectively.

H3K27ac

N	CR	CR-B	Count	Percent
✗	✗	✓	64,479	37.12%
✗	✓	✗	88,368	50.87%
✗	✓	✓	2,672	1.54%
✓	✗	✗	11,123	6.40%
✓	✗	✓	291	0.17%
✓	✓	✗	6,113	3.52%
✓	✓	✓	666	0.38%

H3K4me3

N	CR	CR-B	Count	Percent
✗	✗	✓	125,544	41.99%
✗	✓	✗	56,293	18.83%
✗	✓	✓	6,920	2.31%
✓	✗	✗	91,593	30.64%
✓	✗	✓	7,403	2.48%
✓	✓	✗	3,018	1.01%
✓	✓	✓	8,188	2.74%

H3K9ac

N	CR	CR-B	Count	Percent
✗	✗	✓	1,578	3.91%
✗	✓	✗	37,214	92.29%
✗	✓	✓	129	0.32%
✓	✗	✗	1,238	3.07%
✓	✗	✓	87	0.22%
✓	✓	✗	52	0.13%
✓	✓	✓	24	0.06%
